# Supplementary material for: Evaluating Vitamin D levels in Rheumatic Heart Disease patients and matched controls: A case-control study from Nepal
Source: PLoS One. 2020 Aug 21;15(8):e0237924. doi: 10.1371/journal.pone.0237924 (PMC7444549; doi:10.1371/journal.pone.0237924)
Supplement: S1 File — (DOCX) [file pone.0237924.s002.docx]

**S1 File. Questionnaire used to collect information on demographic variables and for wealth index score analysis.**

**Malnutrition & Rheumatic Heart Disease**

**KTM**

| **Study ID** |  |
| --- | --- |
| **Date** |  |
| **Telephone** |  |

| **Pregnant** |  |
| --- | --- |
| **Oedema** |  |
| **District** |  |

| **RHD Registry** | **Newly Diagnosed RHD** | **Surgical Case** | **Control** |
| --- | --- | --- | --- |
|  |  |  |  |

| **Weight** | **Height** | **MUAC** | **BMI** |
| --- | --- | --- | --- |
|  |  |  |  |

| **IP** |  |
| --- | --- |
| **OPD** |  |
| **Comorbidities** |  |
| **Time since diagnosis** |  |

| **Diagnosis/Echo Report** |
| --- |
|  |

**jfy /f]u ePsf lj/fdLx?df le6fldg l8 / kf]if0fsf] cj:yfaf/] sf] cWoog, sf:sL, kf]v/f**

**Vitamin D and nutritional status among rheumatic heart disease patients in**

- **k|To]s k|Zgx?nfO{ /fd|f] ;Fu k9\g'xf];\ / ;lx hjfkm lbg'xf];\ .**

**Read each question carefully and respond appropriately**

- **olb tkfO{n] s'g} k|Zg j'em\g' ePg eg] ;f]Wg'xf]nf .**

**Feel free to ask if you don't understand/confuse about any question**

- **;lx pQ/sf] sf]7f leq dfq l7s nufpg'xf]nf .**

**Please tick ( X ) or write answer in the box accordingly**

| **k\|+= g+=**  **Q. No.** | **;j]{50f ;DaGwL hfgsf/L**  **Questions** | **pQ/**  **Answer** | |  |
| --- | --- | --- | --- | --- |
| !=  1 | tkfO{+sf] hGdldtL slt xf] <  What is your date of birth? | Bg (Day)============= dlxgf (Month)===========;fn (Year)========= | |  |
| @  2 | tkfO{{ k'?if xf] ls dlxnf xf] <  Are you a male or female? | ☐ dlxnf (Female)  ☐ k'?if (Male) | |  |
| #  3 | tkfO{sf] :s"n s'g k\|sf/sf] xf] <  What is the type of your school? | ☐ ;fj{hlgs (Government)  ☐ Lglh (Private)  ☐ 5}g  (No) | |  |
| $  4 | tkfO{sf] cfdfsf] ;fIf/tf l:ylt s:tf] 5<  What is the literacy status of your mother? | ☐ lg/If/ (Illiterate)  ☐ ;fIf/ (Literate)  ☐ yfxf 5}g (Don’t know) | |  |
| %  5 | tkfO{sf] cfdfsf] z}lIfs of]Uotf slt xf] <  What is the educational level of your mother? | ☐ k\|fylds lzIff-! b]lv %_ (Primary (1-5))  ☐ lgDg dfWolds -^ b]lv *_ (Lower Secondary (6-8))  ☐ dfWolds-( b]lv !)_ (Secondary (9-10))  ☐ pRr dfWolds lzIff-!! b]lv !@_(Higher Secondary (11-12))  ☐ :gfts jf ;f] eGbf dfly (Bachelor and above)  ☐ yfxf 5}g (Don’t know)  ☐ 5}g  (No) | |  |
| ^  6 | tkfO{sf] cfdfsf] d'Vo k]zf s] xf] <  What is the major occupation of your mother? | ☐ lu[x0fL (Housewife)  ☐ s[ifL (Agriculture)  ☐ HofdL (Labor)  ☐ Jofkf/ (Business)  ☐ ;/sf/L hflu/ (Government service)  ☐ u}x\| ;/sf/L hflu/ (Non-government service)  ☐ j}b]lzs /f]huf/ (Foreign employee)  ☐ ljBfyL{ (Student)  ☐ j]/f]huf/ (Unemployed)  ☐ yfxf 5}g (Don’t know)  cGo eP pNn]v ug'{xf]; (Others (specify)) …………………… | |  |
| &  7 | tkfO{sf] slt hgf bfh' efO{ / slt hgf lbbL jlxgL 5g\ <  How many brothers and sisters you have? | …………………………….. bfh'efO{ (Brothers)  …………………………….. lblbjlxgL (Sisters) | |  |
| *   8 | s] tkfO{sf] bfO{, lbbL, efO{ jf cfdf a'jf nfO{ d'6' /f]u nu]sf] 5<  (s[kof pNn]v ug{'xf];\ )  Do any of your brothers, sisters or parents have a heart disease? (please specify who in your family) | ☐ 5, d]/f] (Yes, my) …………………………………………  ☐ 5}g  (No) | |  |
| (   9 | obL 5 eg] s:tf] vfnsf] d'6'/f]u nu]sf] 5<  If yes, what kind of heart disease? | ☐ hGdhft d'6'/f]uL (Born with heart disease)  ☐ jfy Åbo /f]u (Rheumatic heart disease)  ☐ cs}{ k\|sf/sf] (Another type)  ☐ yfxf 5}g (Don’t know) | |  |
| !)   10 | s] tkfO{sf] bfO{, lbbL, efO{ jf cfdf a'jf nfO{ jfy /f]u nu]sf] 5<  Have any of your siblings or parents had rheumatic fever? | ☐ 5, d]/f] (Yes, my) …………………………………………  ☐ 5}g  (No)  ☐ yfxf 5}g (Don’t know) | |  |
| !!  11 | tkfO{sf] kl/jf/ s:tf] k\|sf/sf] xf] <  What is type of your family? | ☐ Psn (Nuclear)  ☐ ;+o'Qm (Joint)  ☐ j[xt (Extended)  ☐ cGo eP pNn]v ug'{xf]; (Others specify)  ……………………………………………………….. | |  |
| !@   12. | uPsf] xKtf cf};tdf lbgdf slt 306f tkfO{n] 6]lnlehg x]g'{ eof] <  During last one week, how many hours you had watched TV per day? | ☐ 5}g  (No)  ☐ Ps lbgdf ! 306f eGbf sd (< 1hour/day)  ☐ Ps lbgdf ! b]lv @ 306f ;Dd (1-2 hours/day)  ☐ Ps lbgdf @ 306f eGbf j9L (> 2hours) | |  |
| !#  13 | s] tkfO{sf] cfkm\g} 3/ 5<  Do you have your own home? | ☐ 5 (Yes)  ☐ 5}g (No) | |  |
| !$  14 | s] tkfO{ cfkm\g} 3/df j:g'x'G5 ls 8]/fdf j:g'x'G5 <  Are you living in your own home or rented house? | ☐ cfkm\g} 3/df (Own)  ☐ 8]/fdf (Rented)  ☐ cGo pNn]v ug'{xf];\ (Others specify) ………………………... | |  |
| !%  15 | tkfO{sf] 3/df ePsf ;fdfg df l7s nufpg'xf];\ <  Tick the items which you have at your home/house | ljh'nL (Electricity)  /]l8of] (Radio)  6]lnlehg (Television)  df]jfOn kmf]g (Mobile phone)  6]lnkmf]g (Telephone)  /]lkm\|h]/]6/ (Refrigerator)  vf6 (Bed) ;f]kmf (Sofa)  b/fh (Cupboard)  sDo'6/ (Computer)  6]jn (Table)  s';L{ (Chair)  38L (Clock)  kª\vf (Fan)  l9sL÷hFftf] (Dhiki/Janto) | ☐ Yes ☐ No  ☐ Yes ☐ No  ☐ Yes ☐ No  ☐ Yes ☐ No  ☐ Yes ☐ No  ☐ Yes ☐ No  ☐ Yes ☐ No  ☐ Yes ☐ No  ☐ Yes ☐ No  ☐ Yes ☐ No  ☐ Yes ☐ No  ☐ Yes ☐ No  ☐ Yes ☐ No  ☐ Yes ☐ No  ☐ Yes ☐ No |  |
| !^  16 | tkfO{sf] 3/df vfgf jgfpgsf] nflu k\|fo u/]/ s'g OGwg k\|of]u x'G5 <  What type of fuel is used mainly for cooking at your home/house? | ljh'nL (Electricity)  Pn lk hL (LPG)  uf]j/ Uof; (Biogas)  dl6\6t]n (Kerosene)  bfp/f (Wood)  u'O7f (Animal dung)  cGo-pNn]v ug'{xf];\_ (Other) | ☐ Yes ☐ No  ☐ Yes ☐ No  ☐ Yes ☐ No  ☐ Yes ☐ No  ☐ Yes ☐ No  ☐ Yes ☐ No  …………………… |  |
| !&  17 | tkfO{sf] 3/sf] 5fgf s]n] jg]sf] 5 <  What is your house’s roof mainly made of? | v/ jf k/fn (Thatched roof)  h:tf kftf (Galvanized sheet)  vk6f (Ceramic stiles)  9nfg u/]sf] (Cement)  cGo-pNn]v ug'{xf];\_ (Others) | ☐ Yes ☐ No  ☐ Yes ☐ No  ☐ Yes ☐ No  ☐ Yes ☐ No  .................................. |  |
| !*  18 | tkfO{+ s]df ;'Tg'x'G5<  What do you sleep on? | knªdf (Bed)  un}rf (Mattress on the floor)  u'G›Ldf (Blanket on the floor)  cGo-pNn]v ug'{xf];\_ (Others) | ☐ Yes ☐ No  ☐ Yes ☐ No  ☐ Yes ☐ No  …………………… |  |
| !(  19 | tkfO{+sf] sf]7fdf slt hgf ;'Tg'x'G5<  How many people sleep in the same room as you? | ………………………………………………………. hgf (People) | |  |
| @)  20 | tkfO{sf] 3/df 3/kfn'jf hgfj/ / s'v'/f 5g\ <  Do you have any livestock, herds, other farm animals or poultry? | ☐ 5 (Yes)  ☐ 5}g (No) | |  |
| @!  21 | Olb 5, s'g s'g hgfj/x? 5g\ <  If yes, please specify which animal/s and how many. | e};L (Buffalo) ………………….  ufO{ (Milk cows or bulls) ………………….  jfv\|f (Goats) ………………….  s'v'/f (Chickens) ……………………………………  xfF; (Ducks) …………………………………………  ;'u'/ (Pigs) ………………………………………….  cGo-pNn]v ug'{xf];\_ (Others specify) | |  |
| @@  22 | tkfO{sf] kl/jf/df oL s'/fx? 5g\ <  Does anyone in the family own Bicycle/rickshaw, Motorcycle/ scooter, three-wheeler, Car, Bus or truck?, Bus or truck? | ;fOsn÷l/S;f (Bicycle/rickshaw)  df]6/;fOsn÷:s'6/(Motorcycle/scooter)  6]Dk' (Three wheeler)  sf/ (Car)  j; jf 6«s (Bus or truck) | ☐ Yes ☐ No  ☐ Yes ☐ No  ☐ Yes ☐ No  ☐ Yes ☐ No  ☐ Yes ☐ No | |

**@# uPsf] xKtfdf tkfO{n] tnsf kmnkm"nx? slt k6s vfg' eof] <**

(23 How many numbers of times you had consumed the following fruits in last one week? )

| **kmnkm"nsf] gfd**  Fruits Name | **gvfPsf]**  Not consumed | **xKtfdf Ps k6s**  Once per week | **xKtfdf @ b]lv $ k6s**  2-4 times week | **xKtfdf % b]lv ^ k6s**  5-6 times week | **^ k6s eGbf j9L**  > 6 times per week |
| --- | --- | --- | --- | --- | --- |
| :ofp  Apple |  |  |  |  |  |
| s]/f  Banana |  |  |  |  |  |
| cDaf  Guava |  |  |  |  |  |
| ;'Gtnf  Orange |  |  |  |  |  |
| c+u'/  Grapes |  |  |  |  |  |
| df;'  Meat |  |  |  |  |  |
| cG8f  Egg |  |  |  |  |  |
| ;f]oljg t]n  Soybean oil |  |  |  |  |  |
| df5f  Fish |  |  |  |  |  |

**tkfO{sf] pQ/x?sf] nflu w]/} w]/} cfef/L 5f} . olb tkfO{sf s]lx ;'emfj 5g\ eg] tn pNn]v ug'{xf];\ .**

**Your responses are greatly appreciated. If you have any other comments, please mention below.**

___________________________________________________________________________________________________________________________________________________________________________________________________________________________________________________________________________________________________________________________________________________________________________________________________________________________________________________________________________________

**wGojfb !!!**

**Thank you!!!**
