# Supplementary material for: Evaluating Vitamin D levels in Rheumatic Heart Disease patients and matched controls: A case-control study from Nepal
Source: PLoS One. 2020 Aug 21;15(8):e0237924. doi: 10.1371/journal.pone.0237924 (PMC7444549; doi:10.1371/journal.pone.0237924)
Supplement: Attachment — Submitted filename: Response to reviewers PLOS ONE 2.docx [file pone.0237924.s005.docx]

Dear Dr. Samson Gebremedhin, editor at PLOS ONE

Thank you for considering this manuscript, PONE-D-20-02934R2 entitled “Evaluating Vitamin D levels in Rheumatic Heart Disease Patients and Matched Controls: A Cross-sectional Study from Nepal”. once again, and for taking the time to comment. We have revised our manuscript accordingly. As requested a version of the manuscript with marked changes as well as a new version without marks have been uploaded alongside this rebuttal letter.

**Additional Editor Comments**

- **In the abstract please state the age range of the subjects.**
  - Thank you, the age range has been added to the abstract as suggested.
- **Background – line 71-73: the sentence needs citation.**
  - The treatment regime is recommended globally and described by the World Heart Federation of which the reference now has been added. The last part about recurrences is based on personal correspondence, why a reference was not added. We have chosen to omit the sentence instead, so no confusion arises. Thank you for pointing our attention to the lack of reference here. Please see changes line 70-72.
- **Background – line 87-95: please merge the two paragraphs.**
  - Thank you. It has been changed in the final version.
- **Methods: please clearly describe (i) how the dietary intake (Table 3) of the study subject was assessed. (ii) how the socioeconomic status was measured and classified.**
  - A new description has been added in the methodological section “collection of sociodemographic information”. The main difference between this study and others alike, is the use of principal components analysis, we believe. A full description of this method can be seen in ref no. 25 “Measuring Equity with Nationally Representative Wealth Quintiles Guide” (please see the Statistical analysis section), but essentially it is based what is currently owned in the household of each individual and is thus giving information of the wealth of the family on a long term basis. This method is more appropriate than basing the SES on income and expenditure information, especially in the context of Nepal, as many have fluctuating and unreliable income patterns. All variables used for the calculations can be seen in Supplement 1, questions 13-17 and 20-22 were used for the SES calculations. but to make it more clear, are also listed here:
    - Own home – rented or bought.
    - Household items: electricity, radio, television, mobile phone, telephone, refrigerator, bed, sofa, cupboard, computer, table, chair, clock, fan, dhiki/janto (a traditional grinding mill in Nepal).
    - Fuel used for cooking: electricity, LPG, biogas, kerosene, wood, animal dung, other.
    - Type of roof on house: thatched, galvanized, ceramic, cement, other.
    - No. of livestock in household: buffalo, milk cow/bull, goat, chicken, duck, pig, other.
    - Ownership of transportation equipment: Bicycle/rickshaw, motorcycle/scooter, three wheeler, car, bus or truck.
  - Similarly the fruit frequency questionnaire used can be seen in supplement 1, question no. 23. The participants ticked boxes of how many times in the last week they had consumed the mentioned items, and based on this, table 3 was created. All fruits were combined in the final table, but in the questionnaire separate fruit items were mentioned, to encourage more accurate memory/information.

Most importantly, all questions have been tailored specifically for Nepal, and have all been used in previous surveys/studies in all areas of the country.
We hope this describes the process sufficiently, and likewise that you will contact us with further questions if not so.

- **Results – Table 1: Please report the p-values for the age categories.**
  - Thank you, it has been added to table 1.
- **Results – Line 208-11: please remove the stratified analysis by sex because the sample size is too low for meaningful sub-sample analysis.**
  - Thank you for pointing this out. The sub-sample analysis have been removed from the result section as asked, as well as from Table 1.
- **Results – Table 2: please clearly designate significant p-values in the table. All the adjusted variables must also be shown in the table.**
  - We have marked the statistically significant p-values with an “*” and listed the adjusted variables in the Table text beneath marked with an “*^a^*”. We hope this is satisfactory and gives a clear overview of the table content.
- **Results – Table 3: the table should have a comprehensive title that describe its content. In the methods section, the authors need to discuss the purpose of the food frequency data analysis.**
  - Thank you for the comment. We agree a purpose is justified. We have added an explanation in the methods section line 134 – 141 elaborating on the purpose behind this questionnaire. To summarize we wanted to characterize the general quality and variance in food consumption in the two groups, as well as investigate whether a difference in vitamin D rich foods were detectable and in concordance with the measured vitamin D concentrations.
- **Results – Figure 3 and 4: As commented earlier, please remove the HFA standard curves from adolescents. The sample size (n=25) is small for any meaningful analysis. Further, as this is a comparative study, merging and describing the two groups into one is practically less meaningful.**
  - After careful consideration, we have come to the same conclusion and removed the section on HFA standard curves from the manuscript. Both in the result section and the discussion. Initially we found the finding quite interesting, but as you have pointed out, the sample size is simply to small. We will keep the finding in mind for possible further investigations. Thank you for making a very good and important point.
- **Please also discuss possible reasons that may alternative explain the association. E.g. less sunlight exposure RHD patients.**
  - Thank you for the comment. We have chosen to incorporate it as a possible explanation for the lower concentrations in the patients and something to look further into for future studies. Please see the discussion line 265-267.
- **Please also discuss that most of the associations are borderline and may not have huge clinical significance. Further, the implication of the small sample size of the study should be discussed.**
  - We understand these very relevant points. A section has been added to the manuscript under the Discussion section line 320-324. We would like the reader to understand that the small association might be explained by the smaller sample size, but of course a larger study might turn out dismissing the associations completely. Outcomes only future studies could clarify.

Thank you for the useful comments and improvement of this manuscript. We are very happy to see it is still up for consideration and hope we have obliged your request in a satisfactory way.

Best Regards,

Lene Thorup
